# Rapid marine oxygen variability: Driver of the Late Ordovician mass extinction

**DOI:** 10.1126/sciadv.abn8345

**Published:** 2022-11-18

**Authors:** Nevin P. Kozik, Seth A. Young, Sean M. Newby, Mu Liu, Daizhao Chen, Emma U. Hammarlund, David P. G. Bond, Theodore R. Them, Jeremy D. Owens

**Affiliations:** ^1^ Department of Earth, Ocean and Atmospheric Science – National High Magnetic Field Laboratory, Florida State University, Tallahassee, FL 32306, USA.; ^2^ Key Laboratory of Cenozoic Geology and Environment, Institute of Geology and Geophysics, Chinese Academy of Sciences, Beijing 100029, China.; ^3^ University of Chinese Academy of Sciences, Beijing 100049, China.; ^4^ Tissue Development and Evolution (TiDE) Division, Department of Laboratory Medicine, Lund University, Lund, Sweden.; ^5^ Department of Geography, Geology and Environment, University of Hull, Hull HU6 7RX, UK.; ^6^ Department of Geology and Environmental Geosciences, College of Charleston, Charleston, SC 29424, USA.

## Abstract

The timing and connections between global cooling, marine redox conditions, and biotic turnover are underconstrained for the Late Ordovician. The second most severe mass extinction occurred at the end of the Ordovician period, resulting in ~85% loss of marine species between two extinction pulses. As the only “Big 5” extinction that occurred during icehouse conditions, this interval is an important modern analog to constrain environmental feedbacks. We present a previously unexplored thallium isotope records from two paleobasins that record global marine redox conditions and document two distinct and rapid excursions suggesting vacillating (de)oxygenation. The strong temporal link between these perturbations and extinctions highlights the possibility that dynamic marine oxygen fluctuations, rather than persistent, stable global anoxia, played a major role in driving the extinction. This evidence for rapid oxygen changes leading to mass extinction has important implications for modern deoxygenation and biodiversity declines.

## Teaser

Thallium isotopes reveal that rapid marine oxygen fluctuations in the Late Ordovician oceans are linked to mass extinction pulses.

## INTRODUCTION

The earliest of the “Big 5” mass extinctions, the Late Ordovician mass extinction (LOME) (*1*, *2*) is classically observed as a two-pulsed extinction that experienced an ~85% loss of all species, making it the second most ecologically severe crisis of the Phanerozoic (*2*–*4*). However, recent studies have identified a long-term decline in marine biodiversity starting in the middle-late Katian, in addition to the smaller-magnitude decreases in biodiversity that occurred around the Katian-Hirnantian boundary and another in the middle Hirnantian (*5*–*8*). Currently, the LOME is the only major mass extinction to be associated with robust geologic evidence for icehouse conditions, and thus, the LOME represents a unique analog for modern climatic feedbacks and serves as an important window to understand biotic responses to rapid climate and environmental change. To date, there is no consensus as to the primary causal mechanism(s) for the LOME; scenarios include rapid global cooling linked to widespread glaciation that created widespread marine habitat loss (*5*, *9*–*12*) and expansion of reducing conditions in the oceans (*13*–*15*) possibly linked to volcanism (*16*).

Some studies have inferred widespread euxinia (anoxic and sulfidic water column) as a causal mechanism for both extinction pulses (*13*, *15*). For example, molybdenum isotope (δ^98^Mo) data suggest regional and potentially global expansions of reducing conditions before and during the early Hirnantian (*13*, *17*) [~445 million years]. Meanwhile, carbonate uranium isotope (δ^238^U) records suggest an increase in the late Hirnantian global anoxic seafloor area (*18*, *19*). However, these studies used abundances and isotopes of trace metals with long modern residence times [e.g., ~450 ka (thousand years) for U and Mo] relative to oceanic mixing time (~1 to 2 ka) that are less likely to capture geologically rapid shifts under oceanic redox conditions (*14*, *15*, *17*, *18*, *20*). Furthermore, these proxies respond to highly reducing conditions, thus limiting their capability to reconstruct fluctuations near the oxic-anoxic boundary (*21*). While these works have documented linkages between reducing conditions and the LOME, the connections between early redox responses and the biotic records from Late Ordovician oceans remain poorly understood.

### Tracking Late Ordovician paleoredox changes

Sedimentary thallium (Tl) isotopes are an emerging paleoredox proxy that has been used to track global Mn oxide burial throughout Earth history (*21*–*28*). The redox potential of Mn is close to O_2_, and thus, it is one of the first elements to respond to changes in marine oxygenation (*29*). Within oxygenated marine conditions, soluble Mn(II) precipitates to insoluble, oxidized Mn(IV) oxides. Under anoxic conditions either in sediments or in the water column, Mn oxides undergo reductive dissolution, releasing dissolved Mn(II) back into the marine reservoir (*30*–*32*). Thallium adsorption onto low-temperature Mn oxides (e.g., birnessite) imparts a large fractionation (*33*, *34*) and is the only flux that can have substantial and short-term effects (see below). Therefore, Tl isotopes can track the global burial of Mn oxides. Furthermore, because of the relatively short modern residence time of Tl (compared to U or Mo, residence time of Tl = ~18.5 ka) (*21*), this proxy can identify rapid fluctuations in marine bottom water paleoredox conditions throughout Earth history. Using this framework, sedimentary Tl isotopic compositions have the potential to provide a more nuanced understanding of the early redox responses to reducing conditions in Late Ordovician oceans.

Modern seawater ε^205^Tl (ε^205^Tl_SW_ = −6.0 ± 0.3) is homogenous and more negative than bulk continental crust (ε^205^Tl = −2.1 ± 0.3) and all inputs (average of ε^205^Tl = −1.7) (*21*, *35*). Mass balance calculations reveal that ε^205^Tl_SW_ is primarily controlled by the sinks of Tl, the alteration of oceanic crust (AOC; ε^205^Tl_AOC_ = −12.0 to −6.0), and burial of Mn oxides (ε^205^Tl_Mn oxides_ = +6.0 to +16.0), as all inputs are isotopically similar (*21*). While the Tl isotopic fractionation between seawater and AOC is small, it represents the largest burial flux (~63%); meanwhile, the burial of Mn oxides has a large isotopic fractionation relative to seawater but represents a smaller flux (~32%) (*21*). Contemporaneous ε^205^Tl_SW_ values are recorded in anoxic and sulfidic sediments, as the last minor sink (~4%), with no isotopic fractionation (*24*, *35*). Over million-year time scales, tectonically driven AOC rates may change baseline ε^205^Tl_SW_ values. However, rapid shifts (<5 Ma) in the Tl isotopic records reported here likely reflect global changes in Mn oxide burial.

To assess whether the authigenic Tl isotopic trends represent contemporaneous seawater values during the time of deposition, local paleoredox conditions must be independently constrained to determine that anoxic to euxinic bottom waters were present. Without these constraints, it is possible that non-negligible local cycling of Mn within a basin could partially or completely overprint global Tl isotopic trends. Core top sediments from the Black Sea and Cariaco basin suggest that euxinic conditions are necessary for quantitative drawdown of Tl and the reliable capture of true seawater values (*35*). In addition, data from the Santa Barbara Basin have suggested that sediments that are deposited under stable anoxic conditions with sulfide limited to the pore fluids faithfully capture overlying Tl isotopic compositions, as long as Mn concentrations are well below the average oxic sediment value of ~850 parts per million (ppm) (*24*). In this study, we present Tl isotope records from two separate and hydrographically unique, peri-equatorial continental margins in the Iapetus and Panthalassic Oceans to elucidate changes in global anoxia associated with the second largest mass extinction in Earth history.

### Geological background

**Fig. 1.**
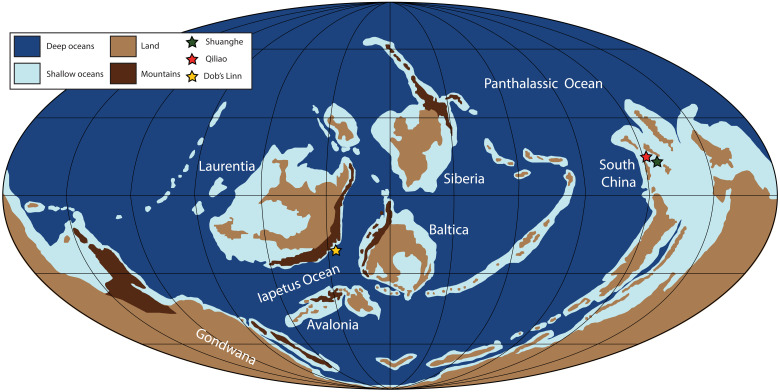
Paleogeographic reconstruction of the Late Ordovician. Paleogeographic reconstruction of the Late Ordovician with study sites denoted by stars (*42*).

Upper Ordovician to lower Silurian strata from Gondwana (South China; Shuanghe and Qiliao sections) and Laurentia (Dob’s Linn, Scotland) represent distal shelf to continental slope settings (Fig. 1). The Shuanghe and Qiliao sections were deposited within the relatively shallow Yangtze Shelf sea that had a direct connection with the Panthalassic Ocean to the north, with the Shuanghe section interpreted to be deposited in a shallower depth than the Qiliao section (*15*, *36*). The Yangtze Shelf sea was primarily a shallow sea that promoted carbonate deposition in the Early and Middle Ordovician but shifted to a siliciclastic-dominated basin in the Late Ordovician during the ongoing accretion of this terrain onto Gondwana (*37*). These two sections from South China have been the subject of previous biostratigraphic and paleoredox studies and contain open marine graptolite (major Paleozoic zooplankton group) fauna allowing for direct correlation to the latest Ordovician Geologic Time Scale 2020 (GTS 2020) (*13*, *38*–*40*). Meanwhile, the Dob’s Linn section is the Global Boundary Stratotype Section and Point for the Ordovician-Silurian boundary, which allows for direct correlation to the GTS 2020 and has been interpreted to be deposited in a continental slope setting on the eastern margin of Laurentia (*41*). This locality represents the deepest marine setting of our study localities presented here because of the limited abundance of sedimentological structures indicative of high-energy environments (*41*, *42*). The Dob’s Linn locality was in direct connection with the Iapetus Ocean as evidenced by the presence of regularly occurring open marine Late Ordovician–early Silurian graptolite faunas, which were used for detailed chronostratigraphic framework (*42*). Furthermore, all three localities have been the subject of previous paleoredox studies, indicating that these sites were deposited under predominantly anoxic to euxinic conditions (*14*, *15*). Specifically, iron speciation data indicate predominantly local anoxic conditions, while mild enrichments of Mo suggest sulfidic porewater conditions to potentially euxinic bottom waters from both South China sections (*15*). The iron speciation paired with Mn and Mo concentrations indicate predominantly anoxic bottom water conditions with potentially intermittent evidence for localized sulfidic porewaters and/or euxinia from the Dob’s Linn section (*14*, *16*). Geologic and geochemical evidence suggests that the Hartfell Shale at the Dob’s Linn section was likely less reducing (dysoxic to anoxic) than the time-equivalent interval of the Wufeng Formation of South China (see the Supplemental Materials for more information). These previous findings support the application of Tl isotopes from each section for reconstructing changes in Mn oxide burial across the LOME interval (see Materials and Methods for further details on sample screening).

## RESULTS

**Fig. 2.**
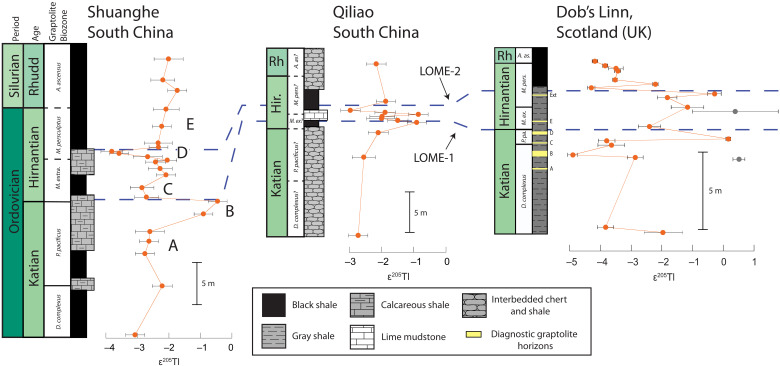
Lithologic and thallium isotopic profiles from South China and Laurentian margins. Intervals A to E denote major changes in Tl isotopic compositions and their correlation (based on graptolite biostratigraphy and carbon isotope chemostratigraphy) to the two classic LOME pulses (blue dashed lines). Gray circles in the Dob’s Linn section represent samples with elevated (Mn) and therefore likely do not represent seawater values. All error bars represent 2σ reproducibility of that sample. Rhudd/Rh, Rhuddanian; Hir, Hirnantian. Yellow-colored bands A to Ext on the Dob’s Linn lithostratigraphic column represent diagnostic graptolite-bearing intervals replotted from Melchin *et al.* (*42*).

Along with our Late Ordovician Tl isotopic datasets, we calculated the durations (highlighted in Fig. 2) for the ε^205^Tl perturbations using linear sedimentation rates for the three localities extrapolated from detailed biostratigraphic studies (*37*, *42*, *43*), high-resolution carbon isotope chemostratigraphy (*15*, *44*), and the most recent Ordovician GTS 2020 (*39*) (see Materials and Methods). Before the Katian-Hirnantian boundary, over a duration of ca. ~900 ka, Tl isotope values shift from initial late Katian values of ε^205^Tl = ~−3.0 to ~−1.0 (Fig. 2, A and B). Subsequently, Tl isotopes return to values of −3.0 in the earliest Hirnantian within ca. ~370 ka (Fig. 2C). This increase in Tl isotope values from the latest Katian through the early Hirnantian coincides with major declines in marine biodiversity observed in several groups, the rising limb of the Hirnantian carbon isotope excursion (*2*), and global sea level fall (*39*). In the mid-Hirnantian, a negative shift in ε^205^Tl is documented, where values decrease from ~−2.0 to ~−4.0 (Fig. 2D), coincident with a global sea level lowstand (*39*) over a duration of ca. ~345 ka. Last, in the late Hirnantian, ε^205^Tl values increase to ~−2.0 (Fig. 2E) over ca. ~150 ka, coincident with a biodiversity low and global sea level rise (*11*, *39*).

To assess the fidelity of our ε^205^Tl trends, geochemical cross plots have been used, and no significant correlation is documented between Mn concentrations and ε^205^Tl values in the Dob’s Linn section (fig. S1). Although these cross plots indicate little to no influence from Mn concentrations at Dob’s Linn, we have conservatively excluded two Tl isotope values (most positive gray data points in Fig. 2). These samples are more likely to be influenced by primary Mn oxides, as they are between Mn-enriched stratigraphic horizons. The Dob’s Linn section has undergone considerable postdepositional heat and pressure, resulting in low-grade metamorphism (*45*), thus the possibility of associated minor geochemical alteration. In addition, there is very weak to no correlation between Fe_HR_/Fe_T_ values and ε^205^Tl values in all three sections (fig. S1), which suggests that there is no connection between local redox and Tl isotope trends. Thus, local early-stage diagenetic processes likely had limited, if any, influence on the overall ε^205^Tl trends documented herein. It is possible, however, that local early stage diagenetic processes may explain the small magnitude (<~0.5 epsilon unit) variability within contemporaneous ε^205^Tl data points between our study sites (i.e., magnifying or dampening the global trend from one section to another). In addition, it is likely that these small variations of Tl isotope compositions between study sites are due to variable sample resolutions for each locality. Sample resolution was dictated by differences in local paleoredox [i.e., sample screening for stable paleoredox conditions with local redox proxies such as total organic carbon (TOC), trace metal concentrations such as Mn, V, Mo, and iron speciation] and/or sedimentation rates within the respective graptolite biozones. Despite local influences, it is unlikely that both paleobasins experienced similar early- and late-stage diagenetic histories to produce similar first-order ε^205^Tl trends apparent in all three sections. Therefore, these records most plausibly reflect changes in the global ocean Tl isotope composition with minimal basinal heterogeneity or restriction affecting ε^205^Tl records.

## DISCUSSION

### Mn oxide burial in Late Ordovician oceans

We conclude that spatiotemporal variations in the loci and extent of Late Ordovician oxygen minimum zones (OMZs) (i.e., shallow/deep shelf versus slope and water column extent) provide a parsimonious model for the fluctuating Mn oxide burial rates needed to generate the two Late Ordovician thallium isotope excursions. The total duration of the geochemical records reported here is slightly longer than other Paleozoic studies that have analyzed sedimentary Tl. Thus, enhanced AOC or weathering-derived Mn inputs have the potential to influence the marine Tl isotopic compositions (*23*, *28*) (see the Supplementary Materials for more details). However, AOC only imparts a small isotopic fractionation relative to seawater, and it is associated with tectonic activity that occurs over multimillion-year time scales (*35*). Given that the observed ε^205^Tl perturbations are large in magnitude, shifting in both positive and negative directions, and occur on submillion–year time scales, large fluctuations in AOC as the primary driver behind the multiple perturbations is unlikely. Enhanced weathering of carbonates has been implicated to explain the Hirnantian δ^13^C_carb_ trends (*9*). Carbonates generally have low concentrations of Tl and Mn and are unlikely to significantly influence ε^205^Tl trends. An increased dissolved marine Mn reservoir, however, may permit increased precipitation of Mn oxides (*46*), but ultimately, this requires excess O_2_ for extensive Mn oxide formation and long-term burial. An enlarged marine Mn reservoir from either enhanced weathering or extensive anoxia could influence the magnitude and duration of ε^205^Tl excursions but does not change the general interpretations. A more reducing global ocean redox state in the Late Ordovician may explain the more positive Tl isotope values in the Katian (ε^205^Tl = −3.0 to −4.0) relative to modern seawater values. This comports with several studies that have indicated that the Paleozoic atmosphere also contained less oxygen than today (*47*, *48*). Models for positive and negative Tl isotope excursions have been published for Cretaceous Oceanic Anoxic Event 2 and the Permian-Triassic boundary (*23*, *35*). Direct comparison of these Tl isotope excursions, however, requires similar source and sink fluxes to estimate global extent. These studies suggest that rapid deoxygenation and oxygenation events, respectively, can produce similar excursions to those present in our Late Ordovician Tl isotope records (*23*, *35*).

### Rapid redox changes during the LOME

**Fig. 3.**
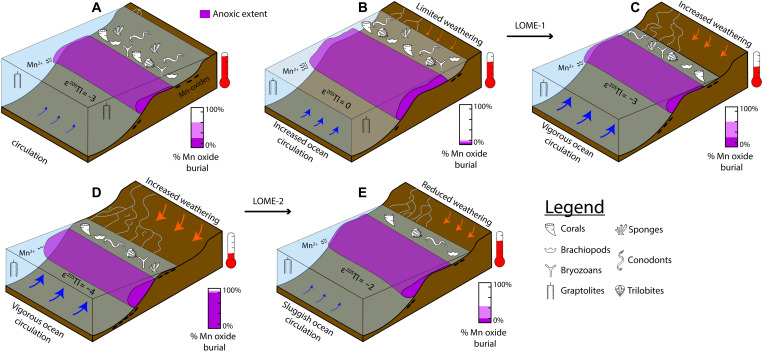
Illustration of idealized latest Ordovician continental margin. Reconstruction of an idealized Late Ordovician margin, where (**A** to **E**) represent corresponding intervals of Tl isotopic change in Fig. 2. Changes in extent of anoxic conditions associated with OMZs (purple water column) are derived from this study. Relative strength of Mn oxide burial at each time slice are shown in purple bar graph, are purely qualitative, and do not represent quantitative flux rates. The abundance of fossil symbols represents relative biodiversity, while black ovals represent Mn oxides in sediments.

**Fig. 4.**
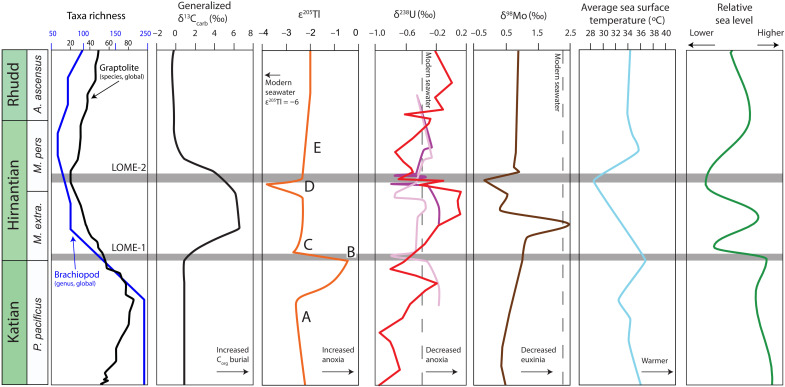
Generalized chemostratigraphic trends of the latest Ordovician and the relationship to biotic change. Compilation of climatic (*11*), physical (*72*), and chemical (*17*–*19*, *73*) paleoceanographic conditions related to the maximum changes in biodiversity associated with the traditional first (LOME-1) and second (LOME-2) extinction pulses (*10*) and recently published graptolite and brachiopod biodiversity trends that are representative Late Ordovician planktonic and benthic groups, respectively (*6*, *7*). Lettering corresponds to changes in ε^205^Tl intervals in Fig. 2. C_org_, organic carbon. δ^238^U is modified from Bartlett *et al.* (*18*) (dark purple line) and reinterpretation presented by Kozik *et al*, (*74*) (light purple line) and Liu *et al.*, (*19*) (red line).

The integration of our Tl isotope data with previously published Late Ordovician palaeontologic data, paleotemperature records, sea level reconstructions, and other paleoredox proxies results in a more holistic assessment of marine environmental conditions during the end-Ordovician biodiversity crisis (Figs. 3 and 4). Before the LOME interval, the Katian was characterized by high sea level (*39*), warm average sea surface temperatures (SSTs) (*12*, *49*), and generally high marine biodiversity until the late Katian when a major decline in biodiversity has been recorded (*6*, *8*, *50*). Elevated SSTs may have reduced thermohaline circulation strength stemming from modest temperature gradients from polar to equatorial regions (*51*). This weaker circulation could have promoted the development of anoxic bottom waters in upper slope to continental shelf settings due to diminished deep-water ventilation, increased remineralization, and reduced oxygen solubility (*52*). The large, positive ε^205^Tl perturbation documented in uppermost Katian strata, before the Katian-Hirnantian boundary, likely indicates an expansion of OMZs on a global scale (Fig. 3, A and B) and is broadly consistent with the first sedimentary indicators of enhanced upwelling (*11*) and falling sea level in the latest Katian (*15*, *53*, *54*). This positive Tl isotope perturbation likely indicates that the initial expansion of oceanic anoxia began in the late Katian, significantly earlier than other records (i.e., trace metal concentration and isotope records) (*2*, *15*, *17*). This expansion of reducing conditions may also be corroborated by U isotopic trends found in South China (*19*). This rapid global expansion of more reducing conditions onto continental shelves and into upper slope settings may have led to the demise of the highly successful and widely geographically dispersed, deep-water *Foliomena-Cyclopyge Fauna* (brachiopod and trilobite fauna, respectively) as well as the decline in various graptolite families (*6*, *7*). Other studies have attempted to correlate the classically defined first LOME pulse at the Katian-Hirnantian boundary to extensive large igneous province (LIP) volcanism and associated warming through the use of enrichments in mercury (Hg) contents and Hg/TOC values from globally dispersed paleocontinents (*16*, *55*–*58*). These geochemical signatures have recently been challenged as Hg anomalies may instead be the product of changes in local marine redox and sedimentary pyrite abundances rather than volcanic loading (*59*). Apart from these geochemical signatures, there is limited geologic evidence for LIP activity surrounding the Katian-Hirnantian boundary, and thus, we do not invoke changes in volcanism/LIP activity as a primary driver for the observed Tl isotopic trends and ultimately the decline in marine biodiversity throughout the end-Ordovician. Instead, we attribute a global expansion of reducing conditions in deep-water settings as a direct kill mechanism for these faunal groups.

Following this first expansion of OMZs on a global scale, comparatively more oxygenated conditions developed in the early Hirnantian oceans, consistent with what would be expected during SST cooling, which is indirectly supported by glacial deposits from Gondwana and records of sea level regression (Fig. 3C) (*60*, *61*). This global sea level fall markedly reduced the total seafloor area available for anoxic bottom water development, coupled with enhanced oxygen solubility and an invigorated thermohaline circulation, resulted in increased oceanic ventilation in the early Hirnantian. Ultimately, the return to lower Tl isotopic values in the earliest Hirnantian likely reflects an increase in Mn oxide burial rates due to enhanced marine bottom-water oxygenation. This early Hirnantian marine ventilation is potentially corroborated by positive δ^98^Mo trends from South China and Dob’s Linn, but the precise timing remains ambiguous because of the coarse resolution of δ^98^Mo from these sections (*13*, *17*). Climatic cooling, intensified thermohaline circulation, and increased gas solubility in early Hirnantian oceans (*12*) are supported by multiple records of sedimentary and geochemical indicators of less-reducing marine conditions from geographically distinct paleocontinents (*42*, *54*, *62*). Our Tl isotope data imply that the initial decline in biodiversity coincided with a rapid initial global expansion of OMZs in the latest Katian and was subsequently followed by a rapid contraction of these OMZs in the early Hirnantian (Fig. 3, B and C). Ultimately, rapid redox fluctuations from the late Katian to early Hirnantian, in combination with widespread marine habitat loss due to glacioeustatic regression, were likely major drivers of the mass extinction that occurred during the end-Ordovician.

Marked and rapid fluctuations in the global marine redox state have also been linked to marine extinction or faunal turnover events in the most severe extinction of the “Big 5”, the end-Permian mass extinction (*23*, *63*, *64*). In the Ordovician, the return to more oxic marine conditions during the Hirnantian could have contributed to the proliferation of survival fauna associated with the LOME [e.g., *Hirnantia* or Edgewood faunas (*2*, *7*)] that may have been better adapted to both cooler SSTs and more variable dissolved oxygen concentrations (*7*). This rapid shift to more oxygenated marine conditions in the early Hirnantian may have also created an additional stressor for organisms adapted to low oxygen conditions.

Following this contraction of more reducing conditions in the early Hirnantian, Tl isotopic compositions increase to −2.0, indicating an expansion of anoxic conditions in the mid-Hirnantian. Another rapid shift (Fig. 3D) to lighter ε^205^Tl values, −2.0 to −4.0, records a transition from widespread reducing conditions to more oxic marine conditions in the mid-late Hirnantian. This minimum in Tl isotopes likely represents a brief ventilation event associated with the highest rates of Mn oxide burial of the latest Ordovician oceans, which likely coincided with the last major glacial advances on Gondwana (*11*, *60*, *61*). Similar to the early Hirnantian, this second marine ventilation event in the mid-late Hirnantian (Fig. 3C) is linked to enhanced thermohaline circulation and decreased SSTs that led to increased gas solubility in the global oceans. This ventilation event is coincident with a global sea level lowstand within the Hirnantian (*39*, *65*, *66*) that further reduced shallow marine habitat space and significantly decreased the total geographic extent of seafloor available for reducing bottom waters to develop along continental margins.

Immediately following this second marine ventilation event in the mid to late Hirnantian, Tl isotopes shift to heavier ε^205^Tl values in all sections, suggesting another interval of rapid changes in marine oxygen contents. This second interval of dynamic redox changes manifested as a rapid oxygenation event (~345 ka), coinciding with a global sea level lowstand and glacial advance. This was immediately followed by a widespread increase in reducing marine conditions (Fig. 3E) and sea level rise associated with the second LOME pulse (*2*, *14*, *15*, *18*). This is consistent with late Hirnantian climatic warming (*11*) and deglaciation, promoting favorable conditions for widespread organic matter–rich shale deposition (*42*) under euxinic conditions, as indicated by recent studies using δ^98^Mo, δ^238^U, and δ^34^S data (Fig. 4) leading into the early Silurian (*14*, *15*, *18*, *19*, *67*, *68*). This final expansion of anoxic conditions (as well as euxinic conditions) likely led to the demise of the survival fauna that flourished in the mid-Hirnantian [e.g, *Hirnantia* faunas (*2*, *7*)]. These data suggest that the rapid marine oxygen fluctuations and feedbacks between global climate and marine paleoredox conditions provide the most parsimonious kill mechanisms for the second LOME pulse (Fig. 4).

Importantly, these results indicate that a combination of environmental factors that include early redox fluctuations, climate variability, and glacioeustacy likely culminated in the second most devastating mass extinction event in Earth history. The rapid expansion of oxygenated marine conditions in the latest Ordovician may have acted as an additional stressor to fauna adapted to reducing conditions; similar scenarios have been documented in the end-Permian (*23*). Meanwhile, persistent and widespread reducing conditions leading into the Silurian are consistent with a protracted and turbulent recovery of marine groups (*8*, *68*). Ultimately, this suggests that the quantitative extent of anoxic conditions alone may have had fewer substantial impacts on Late Ordovician marine life; instead, rapid fluctuations in marine oxygen levels were more devastating. Rapid rates of sea level rise, deglaciation, and increased marine deoxygenation are hallmarks of modern climate change. O_2_ contents have declined by at least 2% over the past 50 years, and models predict continued declines by as much as 7% over the next century (*52*, *69*). The resultant widespread expansion of marine anoxia in the next few thousand years, likely associated with OMZ dynamics, will probably have marked consequences on marine biota and biogeochemical cycles. If anthropogenically induced climate and ocean changes occur at similar rates and durations to environmental variability surrounding the LOME, then this interval may represent a realistic analog to future conditions on Earth.

## MATERIALS AND METHODS

### Tl purification and isotopic analyses

A total of 60 samples between the three sections were selected for Tl analysis. Extraction, purification, and analysis of sedimentary Tl were performed on powdered samples following modified techniques from previous studies (*23*, *26*–*28*). Briefly, samples were leached with 2 M HNO_3_ for 12 hours at 130°C to extract Tl adsorbed to pyrite. The supernatant was subsequently purified using established column chemistry, using micro-columns filled with Bio-Rad AG 1-X8 anion exchange resin to remove all Pb from samples (*23*, *26*–*28*). Purified samples were spiked with NIST-SRM-981 Pb to track mass bias during mass spectrometry using an Aridus II autosampler coupled to a Neptune multicollector–inductively coupled plasma mass spectrometer at the National High Magnetic Field Laboratory at Florida State University. Tl isotopic compositions are compared to the NIST 997 thallium metal standard [reported as ε^205^Tl = (^205/203^Tl_sample_ − ^205/203^Tl_NIST-997_)/^205/203^Tl_NIST-997_ × 10^4^]. Samples were compared to long-term precision of U.S. Geological Survey shale reference material SCo-1, with ε^205^Tl = −3.0 ± 0.3 (2σ). The standard SCo-1 was analyzed for the entire method for each sample batch, and the isotopic values were within the long-term precision. Samples considered for stratigraphic interpretation have 2σ less than 0.5 based on two or more replicate analyses. Any sample with 2σ below the analytical uncertainty of 0.3 had error bars increased to 0.3 because of the uncertainty of the geostandard.

### Sample selection and screening

To assess the extent in which local Mn cycling (e.g., local paleoredox conditions) may be influencing our Tl isotopic trends, we used previous paleoredox studies done on these same stratigraphic successions and samples. Specifically, we utilized paleoredox proxies such as iron speciation and/or Mn concentration datasets and, to a lesser extent, other redox sensitive trace element concentrations such as V, U, and Mo (*14*–*16*). The concentration of Mn in corresponding samples analyzed for Tl is the most powerful tool to assess local cycling of Mn oxides. Where available, we analyzed samples with Mn concentrations of <850 ppm (threshold value for modern oxic marine sediments) (*70*). Mn concentrations were not previously analyzed for the two sections in South China, and we therefore rely on iron speciation and trace metal data that suggest predominantly anoxic to euxinic local paleoredox conditions. Iron speciation from all sections (including Dob’s Linn) indicates pervasive anoxia, and only samples with Fe_HR_/Fe_T_ values that indicate reducing bottom water conditions were considered for Tl isotopic analysis. Last, the mild to moderate enrichments of V, U, and Mo, slightly above upper continental crust values (figs.S2 to S4), indicate persistently reducing sediment pore waters that were reducing and overlain by anoxic to euxinic bottom waters at both South China sections (*71*).

### Sediment accumulation estimates

The duration of each Tl isotopic perturbation was calculated using the latest Ordovician GTS 2020 and absolute ages given therein for biozone boundaries (*39*). Accumulation rates were calculated using these absolute values (GTS 2020 spline ages) for each biozone and subsequently divided by total thickness of each corresponding biozones for all three sections and then averaged. Each perturbation in ε^205^Tl was then calculated from the initial to maximum/minimum values and then back (i.e., rising and/or falling limbs of the two Late Ordovician Tl isotope excursions). For incomplete biozones found in these study localities (e.g., *Dicellograptus complexus* and *Akidograptus ascensus*), we assumed that each location captures the basal or top boundary for each biozone; however, this may cause over/underestimations in the resulting sedimentation rates. Using this framework, the average sedimentation rates from all three sections within the *Pristionchus pacificus*/*Dendropsophu anceps* biozone are ~6 m/Ma; abruptly decrease to <1 m/Ma within the Hirnantian (0.8 and 0.9 m/Ma within the *Metabolograptus extraordinarius* and *Metabolograptus persculptus* biozones, respectively); and subsequently increase to ~2 m/Ma in the earliest Silurian. Average calculated durations for the rising and falling limbs of the first Tl isotope excursion (Fig. 2, B and C) are 908 ± 73 and 371 ± 86 ka, respectively. The average calculated duration for the falling and rising limb of the second Tl isotope excursion (Fig. 2, D and E) are 345 ± 86 and 150 ± 91 ka, respectively. All ages reported here are in thousand years, and errors in these calculations reflect uncertainties in absolute ages in the Ordovician GTS 2020 used to calibrate the duration and absolute ages of the Katian and Hirnantian stages of the Late Ordovician (*39*).
