# Supplementary material for: Rapid marine oxygen variability: Driver of the Late Ordovician mass extinction
Source: Sci Adv. 2022 Nov 18;8(46):eabn8345. doi: 10.1126/sciadv.abn8345 (PMC9674285; doi:10.1126/sciadv.abn8345)
Supplement: Supplementary file 1 — Supplementary Text Figs. S1 to S4 Tables S1 to S3 [file sciadv.abn8345_sm.pdf]

Science Advances
AAAS

Supplementary Materials for

Rapid marine oxygen variability: Driver of the Late Ordovician mass extinction

Nevin P. Kozik *et al.*

Corresponding author: Nevin P. Kozik, nkozik@fsu.edu

Sci. Adv. **8**, eabn8345 (2022)
DOI: 10.1126/sciadv.abn8345

This PDF file includes:

Supplementary Text
Figs. S1 to S4
Tables S1 to S3

Supplementary Text

Additional Influences on Tl isotopes

As stated in the main text, two additional pathways can potentially influence the Tl isotopic trends observed in Upper Ordovician strata: rapid fluctuations in AOC and/or weathering inputs of additional Mn into marine settings. A compilation of active suture lengths, a proxy for potential subduction, throughout the Phanerozoic indicate that a rapid increase in overall suture length occurs prior to Late Ordovician due to Laurentian orogenies and Gondwanan rifting (*75*), thereby increasing the AOC flux. This large increase in orogenic activity has the potential to play a role and explain the negative perturbations observed in our dataset. However, this increase in suture length occurs over multi-million-year timescales and the fractionation associated with AOC is relatively small (~1.7 from seawater). If these changes in AOC were to heavily influence the Tl isotopic changes found in our dataset, then this would require significant changes in spreading rates throughout the analyzed interval of <6 myr and the variability is both positive and negative and on much shorter timescales.

The other potential pathway explored is related to changes in the Mn reservoir size via weathering of crustal material into marine settings. Estimates of weathering from strontium isotopes have placed a ~35% to 60% increase in weathering associated with the Hirnantian carbon isotope excursion (*76*), and it can be assumed that changes in total carbonate weathering represent equivalent increases of dissolved Mn input to marine settings. If this assumption is realistic, a larger dissolved Mn reservoir itself would have limited effect on Tl isotopes, as oxic bottom waters are required to preserve the burial of Mn-oxides in sediments. The adsorption of Tl during Mn-oxide precipitation represents the largest fractionation mechanism for the Tl

system (*18*). If an enlarged dissolved Mn reservoir compared to modern was indeed present in Late Ordovician oceans, then oxic bottom waters would facilitate enhanced formation and burial of Mn-oxides. This larger dissolved Mn reservoir would result in a great capacity for burial, ultimately underestimating the burial rate presented in the main text for the negative excursion. This enlarged reservoir would only impact the magnitude of Tl isotopic compositions, not the direction of Tl isotopic change. While this caveat affects the quantitative burial rate, it does not affect first-order directionality of Tl isotopic perturbations or interpretation of relative oxygenation of the global ocean. Additionally, an enhanced Mn reservoir could also be due to lower Mn-oxide burial rates during times of widespread reducing conditions.

Fig. S1.

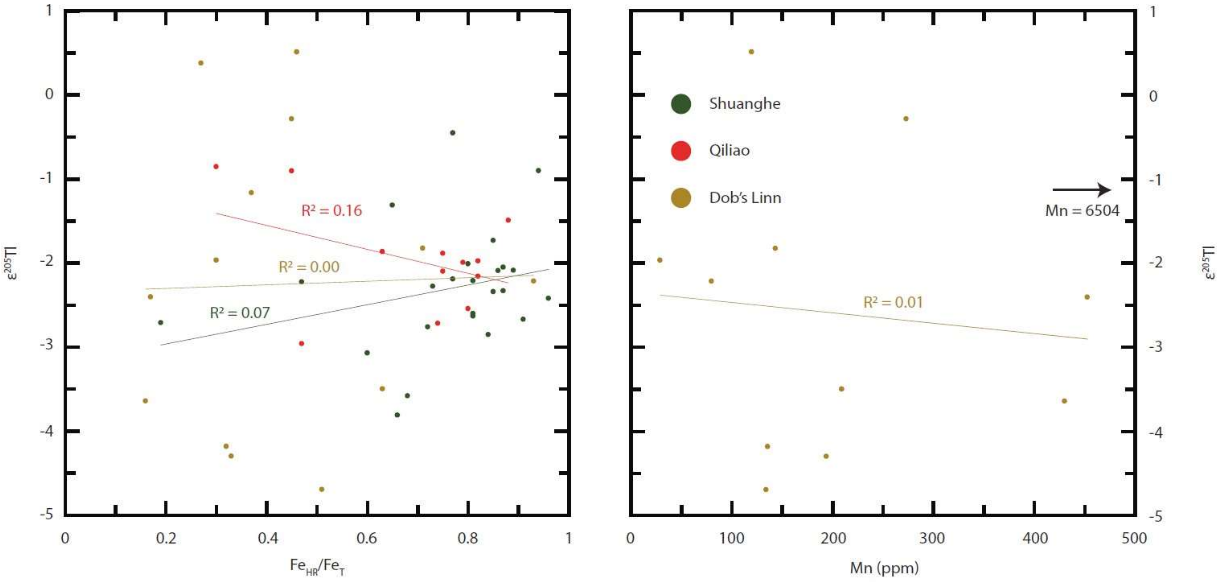

Crossplots of iron speciation vs ε^{205}Tl and Mn concentrations vs ε^{205}Tl. R^2 values show little to no correlation between these geochemical parameters.

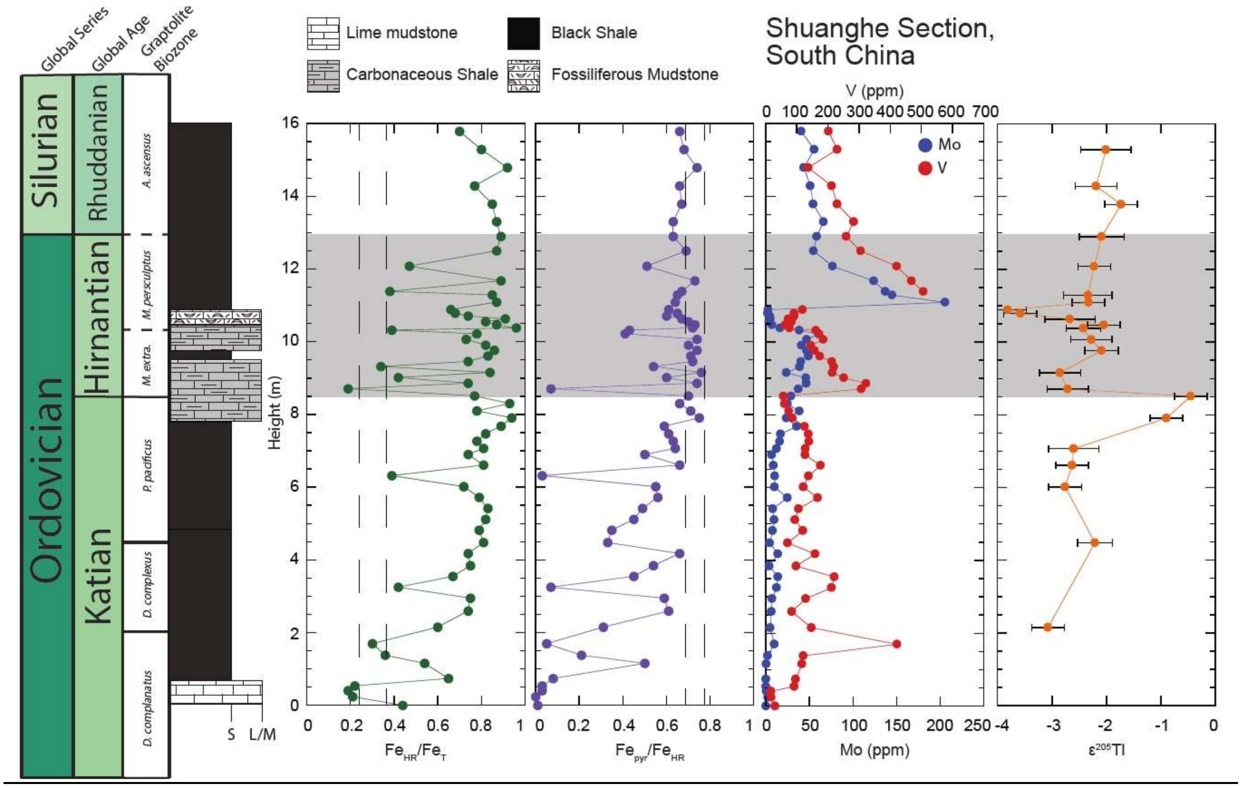

Fig. S2.

Geochemical and lithologic profile of the Shuanghe section, replotted from Zou et al., (*15*).Grey rectangle in all geochemical plots represents the Hirnantian Stage (same for Fig. S2–S4).

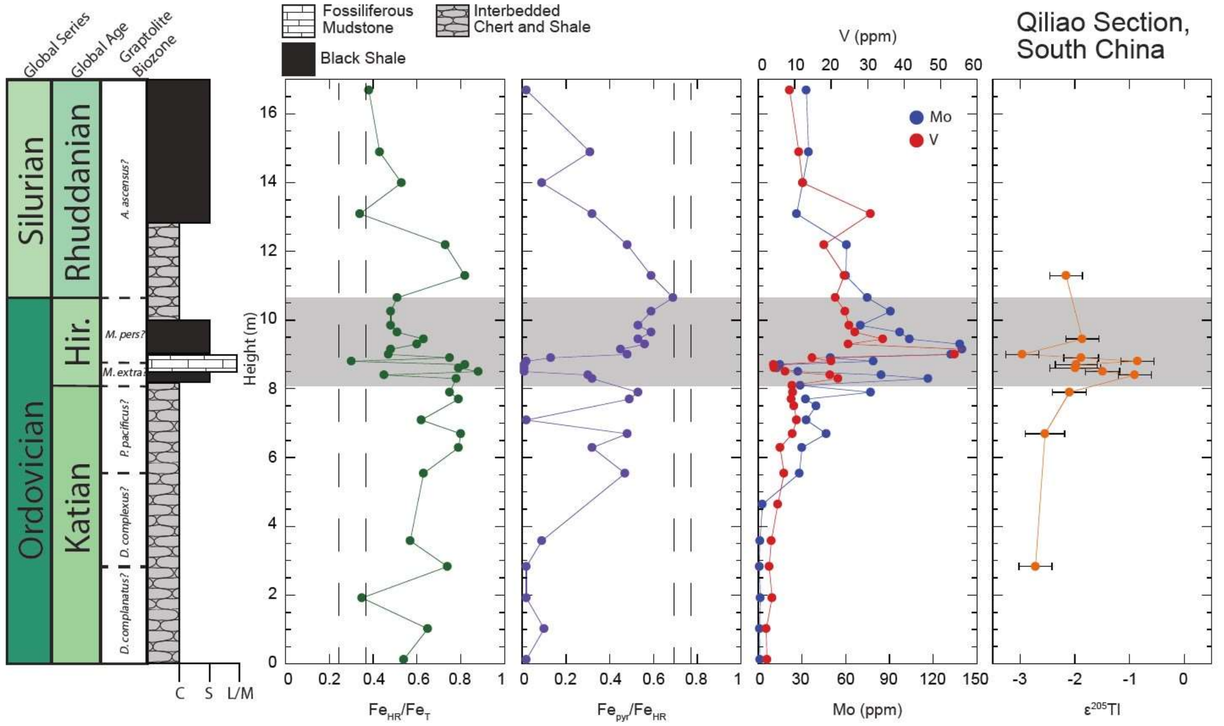

Fig S3.
Geochemical and lithologic profile of the Qiliao section, replotted from Zou et al., (*15*).

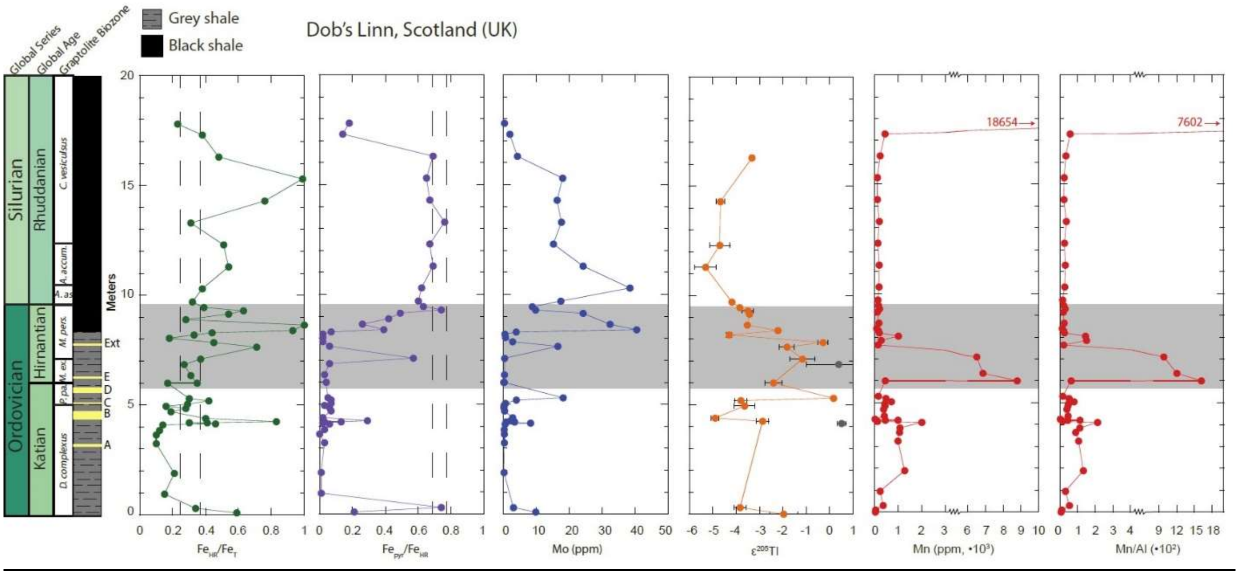

Fig S4.
Geochemical and lithologic profile of the Dob's Linn section, replotted from Hammarlund et al, (*14*). Revised biostratigraphic schemes are utilized from Melchin et al, *(42)*.

Height (m	Section	Sample ID	Graptolite zone	Lithology	ε^{205} Tl	2σ
15.29	Shuanghe	C002	A. ascensus	Calcareous shale	-2.01	0.46
14.29	Shuanghe	C211	A. ascensus	Calcareous shale	-2.19	0.38
13.79	Shuanghe	C210	A. ascensus	Calcareous shale	-1.73	0.30
12.9	Shuanghe	C208	M. persculptus	Calcareous shale	-2.09	0.41
12.08	Shuanghe	C206	M. persculptus	Siliciclastic shale	-2.22	0.26
11.29	Shuanghe	C203	M. persculptus	Calcareous shale	-2.34	0.44
11.09	Shuanghe	C201	M. persculptus	Mudstone	-2.33	0.30
10.89	Shuanghe	C199	M. persculptus	Mudstone	-3.81	0.34
10.79	Shuanghe	C004	M. persculptus	Mudstone	-3.58	0.30
10.63	Shuanghe	C196	M. extraodinarious	Mudstone	-2.67	0.46
10.47	Shuanghe	C194	M. extraodinarious	Mudstone	-2.05	0.30
10.38	Shuanghe	C193	M. extraodinarious	Mudstone	-2.42	0.32
10.07	Shuanghe	C006	M. extraodinarious	Mudstone	-2.28	0.38
9.77	Shuanghe	C187	M. extraodinarious	Mudstone	-2.09	0.31
9.16	Shuanghe	C183	M. extraodinarious	Calcareous shale	-2.85	0.38
8.71	Shuanghe	C180	M. extraodinarious	Calcareous shale	-2.71	0.38
8.51	Shuanghe	C179	P. pacificus	Mudstone	-0.45	0.05
7.91	Shuanghe	C176	P. pacificus	Mudstone	-0.90	0.30
7.07	Shuanghe	C008	P. pacificus	Mudstone	-2.60	0.46
6.61	Shuanghe	C170	P. pacificus	Calcareous shale	-2.63	0.30
6.02	Shuanghe	C168	P. pacificus	Calcareous shale	-2.76	0.30
4.48	Shuanghe	C009	D. complexus	Mudstone	-2.21	0.32
2.15	Shuanghe	C156	D. complexus	Mudstone	-3.07	0.30
0.74	Shuanghe	C150	D. complanatus	Mudstone	-1.31	0.35

Table S1.

Tl isotopic compositions for the Shuanghe section.

Qiliao section

Height (m)	Section	Sample ID	Graptolite Zone	ε^{205}Tl	2σ Tl
11.29	Qiliao	C324	A. ascensus	-2.16	0.36
9.45	Qiliao	C315	M. persculptus	-1.86	0.27
9	Qiliao	C312	M. persculptus	-2.96	0.10
8.9	Qiliao	C311	M. persculptus	-1.89	0.32
8.8	Qiliao	C310	M. persculptus	-0.85	0.17
8.7	Qiliao	C309	M. persculptus	-1.97	0.38
8.6	Qiliao	C308	M. extraodinarious	-1.99	0.46
8.5	Qiliao	C307	M. extraodinarious	-1.49	0.31
8.4	Qiliao	C306	M. extraodinarious	-0.90	0.18
7.9	Qiliao	C301	P. pacificus	-2.10	0.31
6.69	Qiliao	C293	P. pacificus	-2.54	0.36
2.83	Qiliao	C279	D. complexus	-2.72	0.06

Table S2.
Tl isotopic compositions for the Qiliao section.

Dob's Linn

Height (m)	Sample ID	Biozone	Mn (ppm)	ε^{205} Tl	2σ
16.3	DL9	*C. vesiculosus*	231.84	-3.32	0.10
14.3	DL7	*C. vesiculosus*	110.35	-4.68	0.19
12.3	DL5	*A. accuminatus*	134.15	-4.69	0.43
11.3	DL4	*A. accuminatus*	190.96	-5.31	0.46
9.7	DB31	*A. ascensus*	135.63	-4.18	0.07
9.45	DB30	*A. ascensus*	159.34	-3.84	0.06
9.3	DL2	*N. persculptus*	209.04	-3.50	0.24
9.15	DB29	*N. persculptus*	111.76	-3.44	0.10
8.65	DB27	*N. persculptus*	175.57	-3.52	0.08
8.4	DB1	*N. persculptus*	80.03	-2.21	0.09
8.2	DB2	*N. persculptus*	193.76	-4.30	0.12
7.85	DB4	*N. persculptus*	272.93	-0.28	0.22
7.65	DB5	*N. persculptus*	143.30	-1.82	0.32
7.1	DB6	*N. extraordinarius*	6504.33	-1.16	0.53
6.85	DB7	*N. extraordinarius*		0.38	1.37
6	DB 10	*N. extraordinarius*	452.59	-2.40	0.36
5.3	DB11	*P. pacificus*	151.73	0.16	0.05
5.2	DB12	*P. pacificus*	471.51	-3.80	0.27
4.95	DB14	*P. pacificus*	430.13	-3.64	0.43
4.4	DB17	*D. complexus*	406.67	-4.90	0.16
4.25	DB18	*D. complexus*	11.31	-2.87	0.26
4.15	DB21	*D. complexus*	119.69	0.51	0.20
0.3	DB32	*D. complexus*	360.42	-3.83	0.26
-1	DB34	*D. complexus*	28.85	-1.97	0.61

Table S3.

Tl isotopic compositions for the Dob's Linn section.
